# Antibacterial and Anti-Biofilm Activities of Cinnamon Oil against Multidrug-Resistant *Klebsiella pneumoniae* Isolated from Pneumonic Sheep and Goats

**DOI:** 10.3390/pathogens12091138

**Published:** 2023-09-06

**Authors:** Sara H. Mahrous, Farouk A. El-Balkemy, Naser Z. Abo-Zeid, Mamdouh F. El-Mekkawy, Hend M. El Damaty, Ibrahim Elsohaby

**Affiliations:** 1 Infectious Diseases, Department of Animal Medicine, Faculty of Veterinary Medicine, Zagazig University, Zagazig City 44511, Egypt; sarahifny96@gmail.com (S.H.M.); famoawad@vet.zu.edu.eg (F.A.E.-B.); nzmohamed@vet.zu.edu.eg (N.Z.A.-Z.); mfelmekawi@vet.zu.edu.eg (M.F.E.-M.); 2 Department of Infectious Diseases and Public Health, Jockey Club College of Veterinary Medicine and Life Sciences, City University of Hong Kong, Hong Kong SAR 999077, China; 3 Centre for Applied One Health Research and Policy Advice (OHRP), City University of Hong Kong, Hong Kong SAR 999077, China

**Keywords:** *Klebsiella pneumoniae*, sheep and goats, pneumonia, cinnamon oil, antimicrobial, anti-biofilm

## Abstract

The primary objectives were to isolate and identify *Klebsiella pneumoniae* (*K. pneumoniae*), and determine the antimicrobial resistance patterns and biofilm formation abilities of the isolates. Additionally, the study aimed to investigate the antimicrobial and anti-biofilm effects of cinnamon oil against *K. pneumoniae* isolates. A cross-sectional study was conducted from March 2022 to April 2023 to collect 200 samples (including 156 nasal swabs and 44 lung specimens) from pneumonic sheep and goats admitted to the Veterinary Teaching Hospital of Zagazig University, Egypt. *K. pneumoniae* was isolated from a total of 72 (36%) samples, with 53 (73.6%) isolates recovered from nasal swabs and 19 (26.4%) from lung samples. Among the samples, 52 (36.9%) were from sheep and 20 (33.9%) were from goats. Antimicrobial susceptibility testing of the 72 *K. pneumoniae* isolates to 18 antimicrobials revealed that all isolates were resistant to ampicillin, amoxicillin/clavulanic acid, cefotaxime, ceftriaxone, tetracycline, colistin, fosfomycin, and trimethoprim/sulphamethoxazole. None of the isolates were resistant to amikacin, imipenem, and norfloxacin. Multidrug resistance (MDR) was observed in all *K. pneumoniae* isolates recovered from sheep and goats. The average MAR index was 0.71, ranging from 0.50 to 0.83. Regarding biofilm formation, among the *K. pneumoniae* isolates with a high MAR index (n = 30), 10% exhibited strong formation, 40% showed moderate formation, 43.3% displayed weak formation, and 6.7% did not form biofilms. Additionally, the biofilm-forming genes *treC* and *fimA* were present in all 28 biofilm-forming *K. pneumoniae* isolates, while the *mrkA* gene was detected in 15 (53.6%) of the 28 isolates. MDR *K. pneumoniae* isolates with strong biofilm formation abilities were treated with cinnamon oil at varying concentrations (100%, 75%, 50%, and 25%). This treatment resulted in inhibition zone diameters ranging from 35 to 45 mm. Cinnamon oil exhibited lower minimum inhibitory concentration and minimum bactericidal concentration values compared to norfloxacin for all isolates. Additionally, cinnamon oil significantly reduced the expression of biofilm-associated genes (*treC*, *fimA*, and *mrkA*) when compared to isolates treated with norfloxacin or untreated. In conclusion, this study identified a high level of MDR *K. pneumoniae* with strong and moderate biofilm formation abilities in pneumonic sheep and goats in Sharika Governorate, Egypt. Although cinnamon oil demonstrated potential antibacterial and anti-biofilm properties against *K. pneumoniae*, further research is required to investigate its effectiveness in treating *K. pneumoniae* infections in pneumonic sheep and goats.

## 1. Introduction

Small ruminants, such as sheep and goats, play a significant economic role, as they are primarily bred for valuable resources like meat, milk, wool, and hair production, particularly in villages and desert areas, where a considerable portion of the population relies on them [1]. However, ensuring the health of these livestock poses a significant challenge [2]. Pneumonia is a persistent problem that hampers the well-being of small ruminants, causing long-term effects and an overall decline in their quality of life. Its occurrence results from a complex interplay of factors, including host physiology and immunology, various agents such as bacteria, viruses, and parasites, environmental factors, and poor management practices [3]. Prominent symptoms of pneumonic sheep and goats include shallow rapid respiration, inappetence, dyspnea, purulent and mucopurulent nasal discharge, and fever. These symptoms are highly associated with immunosuppression and secondary infection, impacting mortality and morbidity [4].

*Klebsiella pneumoniae* (*K. pneumoniae*) is a virulent bacterium commonly associated with pneumonia in sheep [5,6] and goats [7]. *K. pneumoniae* is receiving increased attention worldwide due to the rise in severe infections, antibiotic resistance, and biofilm formation, presenting challenges for developing effective therapies [8]. Bacterial biofilm development is considered a crucial phase in the pathogenicity of many species [9]. This biofilm protects against phagocytosis, antibody opsonization, and elimination of epithelial cell cilia. Furthermore, biofilm bacteria are significantly more resistant to antibacterial treatments than free-living planktonic cells [10]. *K. pneumoniae* isolates produce biofilms through type 1 and type 3 pili, where the *fimA* and *mrkA* genes encode the major fimbrial subunits, respectively [11]. Additionally, the *treC* gene influences biofilm formation by modulating capsular polysaccharide production. The importance of *treC* in colonization suggests that biofilm formation contributes to the establishment and persistence of *K. pneumoniae* infection [12].

The alarmingly increasing antibiotic resistance among Gram-positive and Gram-negative pathogens necessitates the implementation of alternative treatment strategies. Medicinal plants and their essential oils have demonstrated antibacterial properties against a broad spectrum of virulent bacterial strains in various in vitro experiments. These plants have held economic significance for centuries, serving as a food source and therapeutic agents [13,14]. Cinnamon, in particular, possesses antimicrobial characteristics that suppress the growth of *K. pneumoniae*, and its impact on biofilm formation sets it apart [15,16]. Therefore, this study aims to (i) isolate and identify *K. pneumoniae* from sheep and goats displaying respiratory distress symptoms, (ii) determine the antimicrobial resistance patterns and biofilm formation abilities of *K. pneumoniae* isolates, and (iii) investigate the antimicrobial and anti-biofilm effects of cinnamon oil on *K. pneumoniae* isolates.

## 2. Materials and Methods

### 2.1. Study Design and Sampling

**Figure 1:**
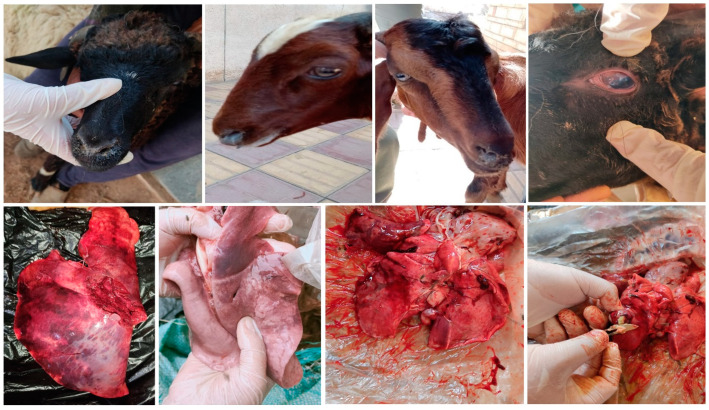
Sheep and goats exhibiting clinical symptoms of pneumonia show mucopurulent nasal discharge with crust formation around the nasal orifice and redness of the ocular mucous membrane (upper row). The lungs of affected sheep and goats display dark-red to grey consolidations and abscesses containing viscous white-yellow odorless pus (lower row).

A one-year cross-sectional study was conducted from March 2022 to April 2023 to isolate *K. pneumoniae* from sheep and goats with respiratory distress symptoms admitted to the Veterinary Teaching Hospital of Zagazig University. Two hundred samples were collected from 141 sheep and 59 goats exhibiting respiratory distress symptoms. The samples included 156 nasal swabs and 44 lung samples with lesions submitted for necropsy examination (Figure 1). Nasal swabs were placed in labeled sterile test tubes containing 3 mL of brain heart broth, and lung specimens were collected in clean and sterile polyethylene bags. The samples were then transported on ice to the laboratory at the Department of Animal Medicine, Zagazig University, Egypt. Animal species, ID, date of collection, districts, age, sex, and type of breeding system were recorded for each animal during the sampling process. A complete clinical assessment and case record for each animal were conducted following the method of Constable et al. [17]. Consent forms were signed by the animal owners before sampling, indicating their agreement to participate in the study.

### 2.2. Isolation and Identification of K. pneumoniae

The collected nasal swabs were incubated at 37 °C for 24 h in brain heart infusion (BHI) broth (Oxoid, Hampshire, UK). However, the lung specimens were homogenized using an automatic grinder instrument (Bertin Technologies, Dieppe, France). The tissue suspensions were centrifuged at 8000× *g* at 4 °C for 10 min and enriched in BHI broth overnight at 37 °C. A loopful from the enrichment broth was streaked onto various selective media, including MacConkey’s agar (Oxoid, Hampshire, UK), eosin methylene blue agar (Oxoid, Hampshire, UK), and HiCrome Klebsiella Selective Agar (Himedia, Mumbai, India). Suspected colonies were purified, Gram stained, and identified as *Klebsiella* spp. using biochemical tests including indole, methyl red, Voges–Proskauer, and citrate, as well as their characteristic reactions on triple sugar iron agar media [18].

### 2.3. DNA Extraction and PCR Amplification

DNA extraction from the suspected *Klebsiella* spp. colonies was performed using the QIAamp DNA Mini kit (Qiagen, Hilden, Germany) following the manufacturer’s instructions. PCR amplifications were carried out in a 25-µL reaction volume using the Applied Biosystems 2720 Thermal Cycler (Foster City, CA, USA). The reaction mixture included 12.5 µL of Emerald Amp Max PCR Master Mix (Takara, Japan), 1 µL of each primer (Metabion, Steinkirchen, Germany) at a concentration of 20 pmoL, 4.5 µL of nuclease-free water, and 6 µL of extracted DNA template. Genus- and species-specific primers for *K. pneumoniae* were used in the PCR amplification (Table 1).

The PCR products were separated by electrophoresis on a 1.5% agarose gel (Applichem, Darmstadt, Germany) prepared in 1X TBE buffer using a voltage gradient of 5 V/cm [21]. For gel analysis, 20 µL of PCR products was loaded into each gel slot, and a 100-bp DNA ladder (Fermentas, Thermo Scientific, Bremen, Germany) was used as a size marker. The gel was photographed using a gel documentation system (Alpha Innotech, Biometra, Göttingen, Germany), and the resulting data were analyzed.

### 2.4. Antimicrobial Susceptibility Testing 

Antimicrobial susceptibility testing of *K. pneumoniae* isolates was performed using the Kirby–Bauer disc diffusion assay on Mueller–Hinton agar medium (Oxoid, Hampshire, UK). A total of 18 antimicrobial agents were tested, including ampicillin (AMP: 10 µg), amoxicillin-clavulanic acid (AMC: 30 µg), gentamycin (GEN: 10 µg), amikacin (AMK: 30 µg), neomycin (NEO: 30 µg), tobramycin (TOB: 10 µg), imipenem (IMP: 10 µg), erythromycin (ERY: 15 µg), cefotaxime (CTX: 30 µg), ceftriaxone (CRO: 30 µg), nalidixic acid (NAL: 30 µg), ciprofloxacin (CIP: 5 µg), norfloxacin (NOR: 10 µg), chloramphenicol (CHL: 30 µg), tetracycline (TET: 30 µg), trimethoprim/sulphamethoxazole (SXT: 25 µg), colistin (CST: 10 µg), and fosfomycin (FOF: 50 µg). The interpretation of inhibition zone diameters was based on the recommendations of the Clinical and Laboratory Standards Institute [22]. Furthermore, the minimum inhibitory concentration (MIC) of colistin (Sigma-Aldrich, Seelze, Germany) was determined for all isolates using the broth microdilution technique following the relevant CLSI instructions [22]. Isolates were classified as multidrug-resistant (MDR) if they were resistant to ≥3 different antimicrobial classes [23]. The multiple antibiotic resistance (MAR) index was calculated for each isolate by dividing the number of antimicrobials to which the isolate displayed resistance by the total number of antimicrobials tested [24].

### 2.5. Biofilm Formation and Quantification

The biofilm-forming ability of *K. pneumoniae* isolates with a high MAR index (n = 30) was assessed using 96-well sterile flat-bottomed polystyrene microtiter plates, following previously described protocol [25]. In brief, an initial bacterial suspension of 10^6^ colony-forming units (CFU)/mL prepared in trypticase soy broth (TSB, Oxoid, Hampshire, UK) was added to each well (200 μL) and incubated at 37 °C for 24 h. The wells were then gently aspirated and washed three times with 200 μL of phosphate-buffered saline (PBS, pH 7.2) to remove any planktonic bacteria. Subsequently, the wells were air-dried for 15 min. Negative control wells containing 200 μL of non-inoculated TSB were included in each test and *K. pneumoniae* ATCC^®^700603™ was used as a positive control.

The biofilm mass was stained with 100 μL of 0.1% crystal violet (Oxoid, Hampshire, UK) for 30 min. After staining, the wells were washed twice with PBS and air-dried. The stained biofilm mass was then re-suspended in an ethanol/acetone solution (80:20, *v*/*v*), and the optical density (OD) was measured at 570 nm (OD_570_) using an ELISA reader (Stat Fax 2100, BioTek, Winooski, VT, USA), with adjustment to the negative control (OD_NC_) to zero. The isolates and negative controls were tested in triplicate. For biofilm quantification, the mean and standard deviation (SD) of the OD values were recorded for *K. pneumoniae* isolates and negative controls. The isolates were classified as none (OD_570_ ≤ OD_NC_), weak (OD_NC_ < OD_570_ ≤ 2 × OD_NC_), moderate (2 × OD_NC_ < OD_570_ ≤ 4 × OD_NC_), or strong (4 × OD_NC_ < OD_570_) biofilm formers.

### 2.6. Detection of Biofilm Forming Genes

The extracted DNA was used for PCR amplification of biofilm-forming genes (*treC*, *fimA*, and *mrkA*) in 28 biofilm-forming *K. pneumoniae* isolates. Conventional PCR assays were performed using the oligonucleotide primer sequences (Table 1) in a 25-µL reaction volume on the Applied Biosystems 2720 Thermal Cycler (Foster City, CA, USA). The reaction mixture consisted of 12.5 µL of Emerald Amp Max PCR Master Mix (TaKaRa Bio Inc., Shiga, Japan), 1 µL of each primer (Metabion, Steinkirchen, Germany) at a concentration of 20 pmoL, 4.5 µL of nuclease-free water, and 6 µL of extracted DNA template. *K. pneumoniae* ATCC^®^700603™ served as a positive control.

### 2.7. Effect of Cinnamon oil on K. pneumoniae

#### 2.7.1. Antibacterial Activity of Cinnamon Oil

The antibacterial activity of cinnamon oil (Sigma-Aldrich, Steinheim, Germany) was evaluated against three strong biofilm-forming *K. pneumoniae* isolates using the agar well diffusion assay [26]. Briefly, fresh cultures of *K. pneumoniae* (1.5 × 10^8^ CFU/mL) were evenly spread on the surface of Mueller–Hinton agar (MHA) plates using a sterile cotton swab. Wells were created in the agar plates using a cork borer with a diameter of 7 mm. Subsequently, under aseptic conditions, the wells were filled with 100 μL of cinnamon oil at four different concentrations (100%, 75%, 50%, and 25%), dissolved in 10% dimethyl sulfoxide (DMSO) obtained from Sigma (Steinheim, Germany). As a negative control, DMSO alone was used. The plates were then incubated at 37 °C for 24 h, following which the diameters of the inhibition zones were measured using a ruler. Each isolate was tested in triplicate.

#### 2.7.2. Minimum Inhibitory Concentration (MIC) Assay

The MIC of cinnamon oil and norfloxacin against three strong biofilm-forming *K. pneumoniae* isolates was determined using a two-fold broth microdilution technique with inoculum size adjusted to approximately 5 × 10^5^ CFU/mL from 0.5 McFarland standard isolates (1.5 × 10^8^ CFU/mL). Stock solutions of cinnamon oil and norfloxacin were prepared at 1024 μg/mL concentration. However, cinnamon oil was diluted with 10% DMSO and norfloxacin was diluted with sterile DW. 

To perform the assay, 100 μL of Mueller–Hinton broth (MHB) was added to each well of the 96-well microtiter plates. Double-fold serial dilutions of the antibacterial agents were prepared by adding 100 μL of the respective agent to the wells. Subsequently, 100 μL of the prepared bacterial inoculum was added to the wells of the microtiter plates. The plates were covered, sealed, and incubated at 37 °C for 24 h. The MIC was determined as the lowest concentration of cinnamon oil and norfloxacin that prevents growth of the microorganism [22]. After incubation, 10 μL aliquots from each well were plated onto MHA plates, which were then incubated at 37 °C for 24 h to determine the minimum bactericidal concentration (MBC) values [27].

#### 2.7.3. Quantitative Analysis of Biofilm Genes Expression

The expression of biofilm-associated genes (*treC*, *mrkA*, and *fimA*) was analyzed in *K. pneumoniae* resistant isolates with strong biofilm formation before and after treatment with cinnamon oil and norfloxacin. Quantitative real-time PCR (qRT-PCR) was performed with 16S rRNA as the housekeeping gene, serving as an internal control to normalize the expression levels among the samples.

RNA extraction was carried out using the RNeasy Mini Kit (Qiagen, Hilden, Germany) following the manufacturer’s instructions. Real-time PCR amplification was performed in triplicate using the Stratagene MX3005P real-time PCR machine (Thermo Fisher, San Jose, CA, USA) and QuantiTect SYBR Green PCR Master Mix (Qiagen, Valencia, CA, USA) according to the manufacturer’s guidelines. To estimate the differences in gene expression among the RNA samples, the threshold cycle (Ct) of each sample was compared to that of the positive control group according to the “∆∆Ct” method [28] using the following ratio: (2^−∆∆ct^).

### 2.8. Data Analysis

Statistical analysis and data visualization were conducted using R software (version 4.2.0). The chi-square (χ^2^) test was utilized to assess the difference in infection rates between sheep and goats. Additionally, a *t*-test was employed to examine the difference in MAR index between isolates obtained from sheep versus goats, as well as between nasal swabs and lung samples. A significance level of <0.05 was used to determine statistical significance.

## 3. Results

### 3.1. Study Population

Sheep and goats admitted to the veterinary teaching hospital of Zagazig University with respiratory distress symptoms such as dyspnea, cough, nasal discharge (purulent and mucopurulent may be tinged with blood), fever, pneumonia, and crusts around the nasal orifice were included in the present study (Figure 1). Of a total of 200 apparently pneumonic animals, 141 (70.5%) were female and 59 (29.5%) were male. The majority of the animals (66.5%) were young, aged one year or younger. The animals were admitted from various localities in Sharkia Governorate: 68 (34%) from Zagazig, 66 (33%) from Abou Hammad, 25 (12.5%) from Abu Kibir, and 41 (20.5%) from Minya Al Qamh. All sheep and goats were indigenous breeds raised for meat production and managed under extensive (68.5%) and semi-intensive (31.5%) production systems. Nasal swabs were collected from 156 animals, comprising 115 sheep and 41 goats. The remaining 44 samples (26 sheep and 18 goats) were lung samples with lesions submitted for necropsy examination (Figure 1). 

### 3.2. K. pneumoniae Isolation and Identification

*K. pneumoniae* was isolated from a total of 72 samples, with 53 (73.6%) isolates recovered from nasal swabs and 19 (26.4%) isolates recovered from lung samples. Among the samples, 52 (36.9%) were from sheep and 20 (33.9%) were from goats. The overall proportion of *K. pneumoniae* infections was 36% (Table 2). There was no significant difference in the infection rate between sheep and goats (*p* = 0.689). However, the infection rate in young animals (≤1 year) was significantly (*p* = 0.028) higher than that in older animals (>1 year).

### 3.3. Antimicrobial Resistance of K. pneumoniae

**Figure 2:**
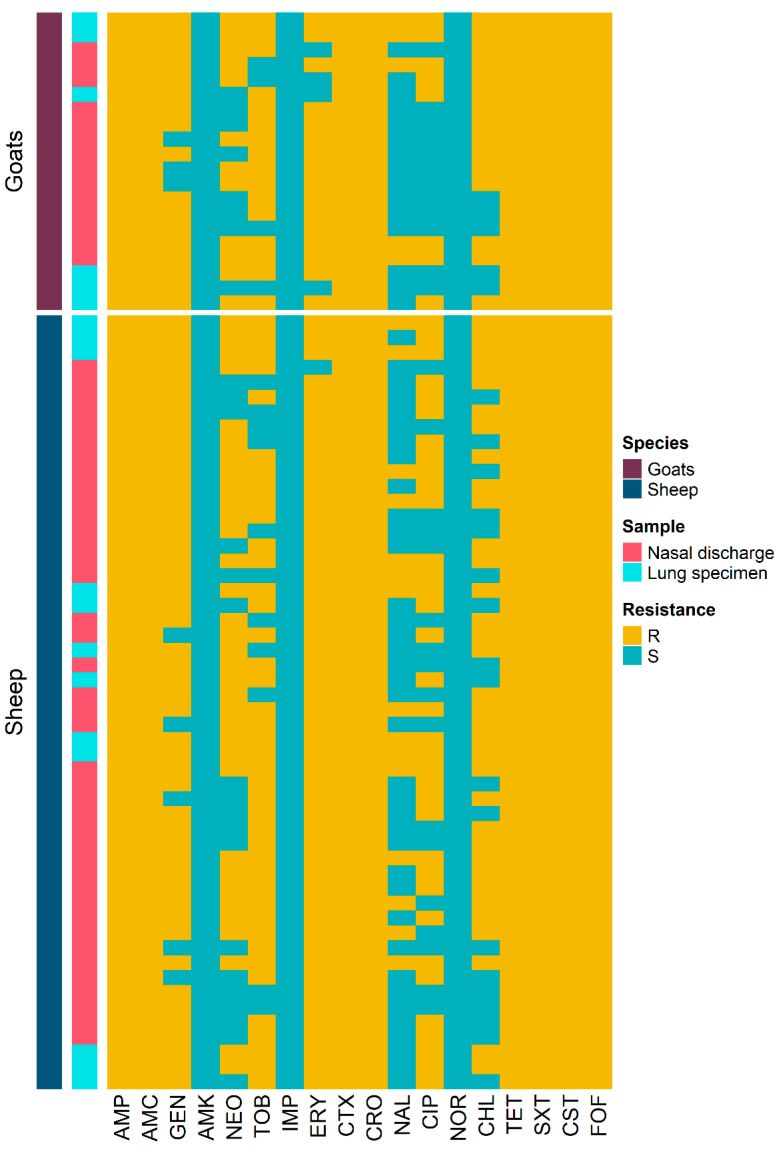
Heatmap representation of the antimicrobial resistance patterns of 72 *K. pneumoniae* isolates recovered from nasal discharge and lung samples of sheep and goats.

Table 3 displays the antimicrobial resistance frequency of the 72 *K. pneumoniae* isolates obtained from sheep and goats against 18 antimicrobials. All isolates demonstrated resistance to AMP, AMC, CTX, CRO, TET, SXT, CST, and FOF (Figure 2). However, none of the isolates were resistant to AMK, IMP, and NOR. All isolates showed resistance to CST, with MIC values ranging from 4 to 128 µg/mL. Multidrug resistance was observed in all *K. pneumoniae* isolates (100%) recovered from sheep and goats. The average MAR index was 0.71, ranging from 0.50 to 0.83. There was no significant difference (*p* = 0.220) in the average MAR index between *K. pneumoniae* isolated from sheep (0.72) and goats (0.69). However, there was a significant difference (*p* = 0.029) in the average MAR index between *K. pneumoniae* recovered from nasal swabs and lung samples. Among the 72 *K. pneumoniae* isolates, 30 (42.9%) displayed a MAR index higher than 0.70. The resistance patterns of these 30 *K. pneumoniae* isolates are presented in Table 4.

### 3.4. Biofilm Formation

The biofilm formation ability of 30 *K. pneumoniae* isolates with a high MAR index was evaluated (Table 4). Among the 30 isolates, 28 (93.3%) isolates exhibited biofilm formation capacity, 3 (10%) isolates displayed strong biofilm formation ability, while 12 (40%) isolates showed moderate ability, and 13 (43.3%) isolates showed weak ability. Only 2 (6.7%) isolates did not form biofilms. The biofilm-forming genes *treC* and *fimA* were detected in all 28 *K. pneumoniae* isolates with biofilm-forming abilities. However, the *mrkA* biofilm-forming gene was found in 15 (53.6%) of the 28 isolates (Table 4).

### 3.5. Effect of Cinnamon Oil on K. pneumoniae

**Figure 3:**
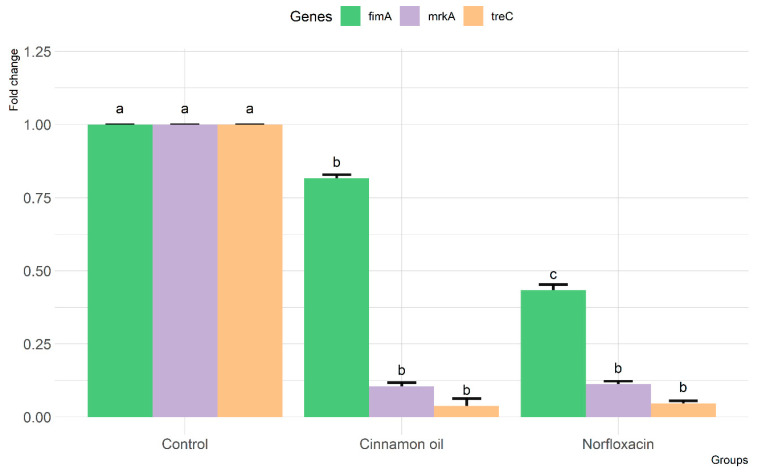
The relative mRNA expression levels of biofilm-forming genes in strong biofilm-forming *K. pneumoniae-*resistant isolates before and after treatment with cinnamon oil and norfloxacin (different letters indicate statistically significant differences).

The disc diffusion method evaluated the zone of microbial growth inhibition using different concentrations of cinnamon oil (100%, 75%, 50%, and 25%). The minimum inhibition zone observed was 35 mm with a 25% concentration, while the maximum zone of inhibition reached 45 mm with 100% cinnamon oil (Supplementary Table S1). The negative control (10% DMSO) showed no growth inhibition. The antimicrobial activity of cinnamon oil was assessed on three *K. pneumoniae* isolates with strong biofilm formation (Table 5). Cinnamon oil demonstrated lower MIC and MBC values compared to norfloxacin for all three isolates. Figure 3 shows the relative gene expression of three biofilm-forming genes (*treC*, *mrkA*, and *fimA*) in *K. pneumoniae* resistant isolates with strong biofilm formation after treatment with cinnamon oil and norfloxacin. Both cinnamon oil and norfloxacin demonstrated a significant decrease in the expression of the three biofilm genes in the treated isolates compared to the non-treated ones.

## 4. Discussion

Small ruminants play a crucial role in the lives and economies of rural populations in Egypt, providing meat, milk, wool, and hair [30]. In this study, 200 animals, consisting of 141 sheep and 59 goats, presenting with respiratory distress symptoms, underwent clinical and bacteriological examination. The results revealed an infection rate of 36% with *K. pneumoniae* in the studied animals. A previous study conducted in Egypt also reported a 36% infection rate of *K. pneumoniae* in pneumonic sheep and goats [31]. However, lower infection rates of 27.15% and 13.39% were reported in small ruminants with respiratory manifestations in Egypt [32] and Nigeria [33], respectively. These variations in infection rates could be attributed to differences in sample size, study population, and epidemiological and ecological characteristics. Nonetheless, it has been established that *K. pneumoniae* is a significant pathogen associated with respiratory problems in less developed countries [32].

In our study, the infection rate of *K. pneumoniae* was slightly higher in sheep than in goats, and in females compared to males. However, these differences did not reach statistical significance, which is consistent with findings reported by Zaghawa and El-Sify [34] and Kattimani et al. [35]. In contrast, recent studies have reported significant differences in the *K. pneumoniae* infection rate based on animal species [36,37] and gender [38]. Animal age was found to be a significant factor affecting the infection rate of *K. pneumoniae*. In our study, we observed a higher infection rate in young animals compared to older ones. This finding is consistent with previous studies that have also reported the susceptibility of young sheep and goats to *K. pneumoniae* infection [35,36,38,39]. The higher susceptibility of young animals can be attributed to factors such as an underdeveloped immune system, increased vulnerability to transportation stress, sudden environmental changes, and viral infections, all of which make them more susceptible to bacterial pneumonia [6,40].

The small ruminants in this study were raised in different localities under extensive and semi-intensive management systems. Although no significant differences were observed in the *K. pneumoniae* infection rate between the localities and management systems, animals raised under the extensive system showed a higher infection rate (38.7%). This higher rate can be attributed to sudden climatic changes, poor nutrition, unhygienic management practices, and the unrestricted contact between animals, which can increase their susceptibility to respiratory and other infections [41].

The antimicrobial susceptibility of the 72 *K. pneumoniae* isolates recovered in this study exhibited varying susceptibility patterns. All isolates were resistant to at least three antimicrobials, and the majority showed resistance to several of the 18 tested antimicrobials. Previous studies on the antimicrobial susceptibility of *K. pneumoniae* isolated from pneumonic sheep and goats have reported different prevalence and patterns [5,31,32,42], which can be attributed to regional variations in antimicrobial use, the availability of over-the-counter antibiotics without prescriptions, and the level of veterinary services provided [32]. In Egypt, over-the-counter availability and extensive use of antimicrobials by smallholder breeders as prophylactics and growth promoters [43,44] may explain the high resistance rate observed in this study.

All *K. pneumoniae* isolates in this study were resistant to penicillins, cephalosporins, tetracycline, sulfonamides, polymyxins, and phosphonic acids, consistent with findings from previous studies in sheep and goats from India [5,42]. Additionally, all recovered isolates were sensitive to amikacin, imipenem, and norfloxacin, likely due to their occasional use in the veterinary field in Egypt. This finding aligns with the results of Ali and Abu-Zaid [31], who reported 100% sensitivity to norfloxacin among *K. pneumoniae* isolates recovered from small ruminants.

All *K. pneumoniae* isolates in this study exhibited MDR and a high MAR index, with most isolates showing high-level resistance to critically important antimicrobial classes such as polymyxins, phosphonic acids, quinolones, macrolides, and aminoglycosides, which are listed by the World Health Organization (WHO) as critically important for human health [29]. The presence of MDR *K. pneumoniae* poses a potential threat to public health, as highlighted by Sen et al. [45].

*K. pneumoniae* isolates capable of forming biofilms pose challenges in treatment as biofilm formation enhances resistance to external stressors and antimicrobials [46,47]. In this study, 93.3% of the 30 *K. pneumoniae* isolates with a high MAR index demonstrated the ability to form biofilms, with 10% exhibiting strong biofilm-forming capacity. Similar findings have been reported in other studies where a high prevalence of *K. pneumoniae* with biofilm-forming capacity was observed in pneumonic sheep and goats [48,49]. Furthermore, a correlation between biofilm formation and antimicrobial resistance was identified in this study.

Interestingly, all biofilm-producing isolates in this study were sensitive to imipenem, which differs from the findings of Mishra et al. [50], who reported higher biofilm-forming capacities in Gram-negative bacteria resistant to meropenem. Moreover, the average MAR index of *K. pneumoniae* isolates with strong and moderate biofilm-forming abilities was higher than that of isolates with weak biofilm formation. This association between biofilm formation and antimicrobial resistance could potentially be attributed to mutations within biofilm-forming genes, which may render biofilm-forming strains sensitive to certain antimicrobials without affecting their ability to form biofilms [51,52]. 

Our study determined the antibacterial activity of cinnamon oil against MDR *K. pneumoniae* isolates with strong biofilm-forming abilities. Cinnamon oil effectively inhibited all tested *K. pneumoniae* isolates at different concentrations. Previous studies have also reported the antibacterial activity of cinnamon oil against *K. pneumoniae* [53,54,55], as well as other bacterial species including *Acinetobacter baumannii*, *Pseudomonas aeruginosa*, *Listeria monocytogenes*, and *Salmonella typhimurium* [56,57]. Cinnamon oil demonstrated higher antimicrobial activity against *K. pneumoniae* compared to norfloxacin, with MIC values ranging from 0.125 to 0.5 μg/mL and MBC values ranging from 0.25 to 1 μg/mL. Similar findings were reported by Wijesinghe, Feiria, Maia, Oliveira, Joia, Barbosa, Boni and HÖfling [15], while Oulkheir, Aghrouch, El Mourabit, Dalha, Graich, Amouch, Ouzaid, Moukale and Chadli [54] reported higher MIC and MBC values of cinnamon oil for *K. pneumoniae*. The antibacterial activity of cinnamon oil can be attributed to its richness in essential oils and tannins, particularly cinnamaldehyde, which inhibits microbial growth [53,58].

Cinnamon oil has been found to be effective in treating *K. pneumoniae* infections by not only exerting antimicrobial effects but also preventing the release of biofilms [16]. Our study investigated the impact of cinnamon oil on the expression of biofilm genes (*treC*, *fimA*, and *mrkA*) in *K. pneumoniae*. The results revealed that cinnamon oil significantly downregulated the expression of these biofilm genes, and its effect was pronounced. Previous studies have also indicated the ability of cinnamon oil to inhibit the expression of biofilm genes in *K. pneumoniae*. For example, Dhara and Tripathi [59] reported that cinnamaldehyde, either alone or in combination with ciprofloxacin, altered the expression of genes encoding porins, efflux pumps, and antibiotic-resistant genes. Additionally, Nuryastuti et al. [60] reported the impact of cinnamon oil on the expression of the *icaA* gene, which is associated with biofilm formation in *Staphylococcus epidermidis*.

One limitation of this study is its focus on a single Egyptian governorate, potentially constraining the applicability of the results to broader regions. Additionally, the study did not explore the in vivo safety of cinnamon oil. A recent study has indicated that higher doses of cinnamon oil could potentially lead to nephrotoxicity and hepatotoxicity. However, it is considered safe when used within the recommended daily dosage [61].

## 5. Conclusions

The present study conducted in Sharkia Governorate, Egypt, revealed a high rate of *K. pneumoniae* infections in pneumonic sheep and goats. Notably, MDR *K. pneumoniae* isolates with strong biofilm-forming abilities were observed in these animals. To effectively control and mitigate the impact of *K. pneumoniae* infection, it is crucial to implement a sustainable control strategy and enhance knowledge and awareness among smallholder breeders. This study highlights the potential of cinnamon oil as an antibacterial and anti-biofilm agent against MDR *K. pneumoniae* isolates with strong biofilm-forming abilities. However, further research is still required to explore the efficacy of cinnamon oil in treating *K. pneumoniae* infections and preventing biofilm formation in pneumonic sheep and goats.

## Figures and Tables

**Table 1:** Oligonucleotide primer sequences, product size, and annealing temperatures used in the present study.

Target Genes	Nucleotide Sequence (5′→3′)	Amplified Segment (bp)	References
Bacterial identification			
*Klebsiella* species *(gyrA*)	F: CGCGTACTATACGCCATGAACGTA	441	[19]
	R: ACCGTTGATCACTTCGGTCAGG		
*K. pneumonia (16S-23SITS)*	F: ATTTGAAGAGGTTGCAAACGAT	130	[20]
	R: TTCACTCTGAAGTTTTCTTGTGTTC		
Biofilm genes			
*treC*	F: CCGACAGCGGGCAGTATT	71	[12]
	R: CGCCGGATTCTCCCAGTT		
*fimA*	F: CGGACGGTACGCTGTATTTT	500	[11]
	R: GCTTCGGCGTTGTCTTTATC		
*mrkA*	F: CGGTAAAGTTACCGACGTATCTTGTACTG	498	
	R: GCTGTTAACCACACCGGTGGTAAC		

**Table 2:** Demographic characteristics of the study population.

Variables	Categories	Sheep (N = 141)		Goats (N = 59)		Total Number of Infected Animals (%)
		No.	Infected (%)	No.	Infected (%)	
Gender						
	Female	99	38 (38.4)	42	14 (33.3)	52 (36.9)
	Male	42	14 (33.3)	17	6 (35.3)	20 (33.9)
Age						
	≤1 year	97	38 (39.2)	36	17 (47.2)	55 (41.4)
	>1 year	44	14 (31.8)	23	3 (13.0)	17 (25.4)
Raising systems						
	Extensive	97	37 (38.1)	40	16 (40.0)	53 (38.7)
	Semi-intensive	44	15 (34.1)	19	4 (21.1)	19 (30.2)
Season						
	Winter	69	22 (31.9)	29	9 (31.0)	31 (31.6)
	Spring	44	17 (38.6)	19	8 (42.1)	25 (39.7)
	Autumn	18	7 (38.9)	8	2 (25.0)	9 (34.6)
	Summer	10	6 (60.0)	3	1 (33.3)	7 (53.9)
Localities						
	Zagazig	59	24 (40.9)	9	6 (66.7)	30 (44.1)
	Minya Al Qamh	10	3 (30.0)	31	6 (19.4)	9 (21.9)
	Abu Kibir	19	9 (47.4)	6	5 (83.3)	14 (56.0)
	Abou Hammad	53	16 (30.2)	13	3 (23.1)	19 (28.8)

**Table 3:** Frequency of *K. pneumoniae* resistant isolates recovered from sheep and goats.

Rank ^1^	Class	Agent ^2^	No. of Resistant *K. pneumoniae* Isolates (%)		
			N = 72	Sheep (n = 52)	Goats (n = 20)
I	Aminoglycosides	AMK	0 (0.0)	0 (0.0)	0 (0.0)
		GEN	64 (88.9)	47 (90.4)	17 (85.0)
		NEO	46 (63.9)	34 (65.4)	12 (60.0)
		TOB	57 (79.2)	41 (78.9)	16 (80.0)
I	Carbapenems	IMP	0 (0.0)	0 (0.0)	0 (0.0)
I	Macrolides	ERY	67 (93.1)	51 (98.1)	16 (80.0)
I	Cephalosporins	CTX	72 (100)	52 (100)	20 (100)
		CRO	72 (100)	52 (100)	20 (100)
I	Quinolones	NAL	20 (27.8)	15 (28.9)	5 (25.0)
		CIP	43 (59.7)	35 (67.3)	8 (40.0)
		NOR	0 (0.0)	0 (0.0)	0 (0.0)
I	Polymyxins	CST	72 (100)	52 (100)	20 (100)
I	Phosphonic acids	FOF	72 (100)	52 (100)	20 (100)
II	Penicillins	AMP	72 (100)	52 (100)	20 (100)
		AMC	72 (100)	52 (100)	20 (100)
II	Amphenicols	CHL	49 (68.1)	34 (65.4)	15 (75.0)
II	Tetracycline	TET	72 (100)	52 (100)	20 (100)
II	Sulfonamide	SXT	72 (100)	52 (100)	20 (100)

^1^ Rank I, critically important; rank II, highly important (based on the World Health Organization’s categorization [29]). ^2^ AMP: ampicillin; AMC: amoxicillin/clavulanic acid; AMK: amikacin; GEN: gentamycin; NEO: neomycin; TOB: tobramycin; IMP: imipenem; ERY: erythromycin; CTX: cefotaxime; CRO: ceftriaxone; NAL: nalidixic acid; CIP: ciprofloxacin; NOR: norfloxacin; CHL: chloramphenicol; TET: tetracycline; SXT: trimethoprim/sulphamethoxazole; CST: colistin; FOF: fosfomycin.

**Table 4:** Antimicrobial resistance profiles, biofilm-forming capacity, and genes of *K. pneumoniae* resistant isolates recovered from sheep and goats.

ID	Source	Antimicrobial Resistance Patterns ^1^	MAR Index ^2^	Biofilm	
				Capacity	Genes
2	Sheep	AMP, AMC, GEN, NEO, TOB, ERY, CTX, CRO, NAL, CIP, CHL, TET, SXT, CST, FOF	0.83	Strong	*treC*, *mrkA*, *fimA*
3	Sheep	AMP, AMC, GEN, NEO, TOB, ERY, CTX, CRO, CIP, CHL, TET, SXT, CST, FOF	0.78	Weak	*treC*, *fimA*
5	Goat	AMP, AMC, GEN, NEO, TOB, ERY, CTX, CRO, NAL, CIP, CHL, TET, SXT, CST, FOF	0.83	Moderate	*treC*, *mrkA*, *fimA*
6	Sheep	AMP, AMC, GEN, NEO, TOB, ERY, CTX, CRO, NAL, CIP, CHL, TET, SXT, CST, FOF	0.83	Moderate	*treC*, *mrkA*, *fimA*
9	Sheep	AMP, AMC, GEN, NEO, TOB, ERY, CTX, CRO, NAL, CIP, CHL, TET, SXT, CST, FOF	0.83	Moderate	*treC*, *mrkA*, *fimA*
36	Sheep	AMP, AMC, GEN, NEO, TOB, ERY, CTX, CRO, CIP, CHL, TET, SXT, CST, FOF	0.78	Weak	*treC*, *fimA*
37	Sheep	AMP, AMC, GEN, NEO, TOB, ERY, CTX, CRO, NAL, CIP, TET, SXT, CST, FOF	0.78	Weak	*treC*, *fimA*
38	Sheep	AMP, AMC, GEN, NEO, TOB, ERY, CTX, CRO, CIP, CHL, TET, SXT, CST, FOF	0.78	Weak	*treC*, *fimA*
39	Sheep	AMP, AMC, GEN, NEO, TOB, ERY, CTX, CRO, NAL, CIP, CHL, TET, SXT, CST, FOF	0.83	Moderate	*treC*, *mrkA*, *fimA*
43	Sheep	AMP, AMC, GEN, NEO, TOB, ERY, CTX, CRO, NAL, CIP, CHL, TET, SXT, CST, FOF	0.83	Moderate	*treC*, *mrkA*, *fimA*
52	Sheep	AMP, AMC, GEN, NEO, TOB, ERY, CTX, CRO, NAL, CIP, CHL, TET, SXT, CST, FOF	0.83	Moderate	*treC*, *mrkA*, *fimA*
55	Sheep	AMP, AMC, NEO, TOB, ERY, CTX, CRO, CIP, CHL, TET, SXT, CST, FOF	0.72	None	--
61	Goat	AMP, AMC, GEN, NEO, ERY, CTX, CRO, NAL, CIP, CHL, TET, SXT, CST, FOF	0.78	Weak	*treC*, *fimA*
72	Sheep	AMP, AMC, GEN, NEO, TOB, ERY, CTX, CRO, CIP, TET, SXT, CST, FOF	0.72	None	--
76	Sheep	AMP, AMC, GEN, NEO, TOB, ERY, CTX, CRO, NAL, CIP, CHL, TET, SXT, CST, FOF	0.83	Moderate	*treC*, *mrkA*, *fimA*
114	Sheep	AMP, AMC, GEN, NEO, TOB, ERY, CTX, CRO, NAL, CIP, CHL, TET, SXT, CST, FOF	0.83	Moderate	*treC*, *mrkA*, *fimA*
115	Sheep	AMP, AMC, GEN, NEO, TOB, ERY, CTX, CRO, NAL, CIP, CHL, TET, SXT, CST, FOF	0.83	Moderate	*treC*, *mrkA*, *fimA*
118	Sheep	AMP, AMC, GEN, NEO, TOB, ERY, CTX, CRO, NAL, CIP, CHL, TET, SXT, CST, FOF	0.83	Moderate	*treC*, *mrkA*, *fimA*
134	Goat	AMP, AMC, GEN, NEO, TOB, ERY, CTX, CRO, NAL, CIP, CHL, TET, SXT, CST, FOF	0.83	Strong	*treC*, *mrkA*, *fimA*
135	Sheep	AMP, AMC, GEN, NEO, TOB, ERY, CTX, CRO, NAL, CIP, CHL, TET, SXT, CST, FOF	0.83	Moderate	*treC*, *mrkA*, *fimA*
136	Sheep	AMP, AMC, GEN, NEO, TOB, ERY, CTX, CRO, NAL, CIP, CHL, TET, SXT, CST, FOF	0.83	Moderate	*treC*, *mrkA*, *fimA*
144	Sheep	AMP, AMC, GEN, NEO, TOB, ERY, CTX, CRO, CIP, CHL, TET, SXT, CST, FOF	0.78	Weak	*treC*, *fimA*
145	Sheep	AMP, AMC, GEN, NEO, TOB, ERY, CTX, CRO, CIP, CHL, TET, SXT, CST, FOF	0.78	Weak	*treC*, *fimA*
146	Sheep	AMP, AMC, GEN, NEO, TOB, ERY, CTX, CRO, NAL, CHL, TET, SXT, CST, FOF	0.78	Weak	*treC*, *fimA*
147	Sheep	AMP, AMC, GEN, NEO, TOB, ERY, CTX, CRO, CIP, CHL, TET, SXT, CST, FOF	0.78	Weak	*treC*, *fimA*
148	Sheep	AMP, AMC, GEN, NEO, TOB, ERY, CTX, CRO, NAL, CHL, TET, SXT, CST, FOF	0.78	Weak	*treC*, *fimA*
150	Sheep	AMP, AMC, GEN, NEO, TOB, ERY, CTX, CRO, NAL, CIP, CHL, TET, SXT, CST, FOF	0.83	Strong	*treC*, *mrkA*, *fimA*
170	Sheep	AMP, AMC, GEN, NEO, TOB, ERY, CTX, CRO, CIP, CHL, TET, SXT, CST, FOF	0.78	Weak	*treC*, *fimA*
175	Sheep	AMP, AMC, GEN, NEO, TOB, ERY, CTX, CRO, CIP, CHL, TET, SXT, CST, FOF	0.78	Weak	*treC*, *fimA*
198	Goat	AMP, AMC, GEN, NEO, TOB, ERY, CTX, CRO, CIP, CHL, TET, SXT, CST, FOF	0.78	Weak	*treC*, *fimA*

^1^ AMP: ampicillin; AMC: amoxicillin/clavulanic acid; AMK: amikacin; GEN: gentamycin; NEO: neomycin; TOB: tobramycin; IMP: imipenem; ERY: erythromycin; CTX: cefotaxime; CRO: ceftriaxone; NAL: nalidixic acid; CIP: ciprofloxacin; NOR: norfloxacin; CHL: chloramphenicol; TET: tetracycline; SXT: trimethoprim/sulphamethoxazole; CST: colistin; FOF: fosfomycin; ^2^ MAR index: multiple antibiotic resistance index.

**Table 5:** Minimum inhibitory concentration (MIC) and minimum bactericidal concentration (MBC) of cinnamon oil and norfloxacin against strong biofilm-forming *K. pneumoniae-*resistant isolates recovered from sheep and goats.

Isolate ID	Source	Sample	MIC (μg/mL)		MBC (μg/mL)	
			Cinnamon Oil	Norfloxacin	Cinnamon Oil	Norfloxacin
2	Sheep	Lung specimen	0.125	32	0.25	64
134	Goat	Nasal discharge	0.5	16	1	32
150	Sheep	Nasal discharge	0.25	8	0.5	16

## Data Availability

The data presented in this study are available on request from the corresponding authors.

## Supplementary Materials

The following supporting information can be downloaded at: https://www.mdpi.com/article/10.3390/pathogens12091138/s1, Table S1: Antibacterial activity of cinnamon oil.
