# Supplementary material for: Antibacterial and Anti-Biofilm Activities of Cinnamon Oil against Multidrug-Resistant Klebsiella pneumoniae Isolated from Pneumonic Sheep and Goats
Source: Pathogens. 2023 Sep 6;12(9):1138. doi: 10.3390/pathogens12091138 (PMC10536549; doi:10.3390/pathogens12091138)
Supplement: Supplementary file 1 [file pathogens-12-01138-s001.zip › pathogens-2543725-supplementary.pdf]

Supplementary Table S1: Antibacterial activity of cinnamon oil

ID	Inhibition zone of cinnamon oil (mm) at different concentrations			
	**25%**	**50%**	**75%**	**100%**
**Isolate 1**	43	43	45	45
**Isolate 2**	40	43	44	45
**Isolate 3**	35	39	40	40
